# Face masks reduce interpersonal distance in virtual reality

**DOI:** 10.1038/s41598-022-06086-x

**Published:** 2022-02-09

**Authors:** Leon O. H. Kroczek, Stephanie Böhme, Andreas Mühlberger

**Affiliations:** 1 Department of Psychology, Clinical Psychology and Psychotherapy, Universität Regensburg, Regensburg, Germany; 2 Department of Psychology, Clinical Psychology and Psychotherapy, Friedrich-Alexander-Universität Erlangen-Nuremberg, Erlangen, Germany

**Keywords:** Psychology, Human behaviour

## Abstract

During the COVID-19 pandemic several behavioral measures have been implemented to reduce viral transmission. While these measures reduce the risk of infections, they may also increase risk behavior. Here, we experimentally investigate the influence of face masks on physical distancing. Eighty-four participants with or without face masks passed virtual agents in a supermarket environment to reach a target while interpersonal distance was recorded. Agents differed in wearing face masks and age (young, elderly). In addition, situational constraints varied in whether keeping a distance of 1.5 m required an effortful detour or not. Wearing face masks (both self and other) reduced physical distancing. This reduction was most prominent when keeping the recommended distance was effortful, suggesting an influence of situational constraints. Similarly, increased distances to elderly were only observed when keeping a recommended distance was effortless. These findings highlight contextual constraints in compensation behavior and have important implications for safety policies.

## Introduction

The COVID-19 pandemic challenges human behavior in an unprecedented manner. In order to reduce viral spreading, several measures have been suggested, namely, physical distancing, hygiene measures, and wearing face masks. While these measures are effective in reducing SARS-CoV-2 transmission, it has been emphasized that single measures do not provide full protection against an infection^1,2^. Instead, the combination of measures, such as physical distancing and wearing face masks, is most effective in reducing infections^3^. The combination of measures, however, might lead to unwanted risk compensation behavior, thereby posing a threat to the containment of COVID-19^4^. Risk compensation theories suggest that the introduction of safety measures is often accompanied by increased risk behavior^5^. Risk compensation has been related to health and safety interventions. For instance, it has been suggested that wearing helmets increases risky driving in cyclists^6^ (but see^7^). Similar mechanisms might influence risk behavior and rule adherence in the COVID-19 pandemic. More specifically, wearing of face masks might reduce adherence to physical distance recommendations. This is supported by data from a previous study showing increased mobility patterns after the introduction of mask mandates in the USA^8^. In contrast, a study in Germany found increased compliance with other safety measures after the implementation of a mandatory mask policy^9^. Importantly, both studies used correlational designs. In order to investigate causal relations between mask wearing and IPD behavior, however, experimental studies are required. Experimental evidence might allow to understand behavioral effects that reduce adherence to policies and is therefore important for an efficient implementation of safety measures.

Interpersonal distance (IPD) is not only a major measure against viral transmission but also a powerful social cue that allows to coordinate behavior and relations on a habitual and mainly unconscious level^10^. Distance between persons is influenced by socio-cultural and psychological factors, including gender, age, relation, and emotions^11,12^. While small IPDs are perceived as arousing, threatening, and are typically avoided in interactions with strangers^13,14^, an optimal IPD around 1 m has been suggested for social interactions^15^. Importantly, this distance is smaller than the common recommendation of a minimal distance of 1.5 m to reduce SARS-CoV-2 transmission^16,17^. Thus, following distance recommendations requires behavioral adaptation via self-control and might be challenging for some people. Biehl et al. (2021) showed that elderly have difficulties in calling for the requested minimal distance when they are approached by other people^18^. Furthermore, interpersonal distance behavior is largely dependent on culture^11^ and it could be demonstrated that country-specific preferences of interpersonal distance and touch behavior were positively correlated with early dynamics of SARS-CoV-2 spread^19^. Covid-19 transmission was higher in countries with preferences for shorter interpersonal distances. Consequently, factors influencing IPD directly relate to the success of physical distancing in COVID-19 containment.

Recent studies have been using online computer-paradigms to evaluate the influence of face masks on IPD. Cartaud et al. (2020) found that shorter distances towards virtual characters were perceived as more appropriate when characters were displayed with face masks compared to characters without face masks^20^. Similar effects were observed in other online studies where participants preferred shorted distances to characters with face masks compared to characters without face masks^21–23^. In addition, Luckman et al. (2020) found that both own and other face masks reduced distance preferences in interactive settings. Crucially, these studies inferred IPD behavior from ratings using projective paradigms rather than measure actual behavior in social interactions ^20,23^. In contrast, Seres et al. (2020) measured IPD in real-life outdoor waiting lines and found that persons kept greater distances to a confederate with a face mask compared to a confederate without face mask^24^. These conflicting results might be related to differences in socio-emotional and physical settings between real-life situations and online experiments. According to Seres et al. (2020), participants associated face masks with a person’s wish for greater IPD and thus increased distances due to politeness. In online experiments, however, participants may not have attributed such motives to virtual characters leading to differing results. Finally, outdoor waiting lines are typically less constrained in space so that distance rules can be easily followed. Different patterns may be observed, when adherence to distance rules is difficult due to situational constraints, for instance in supermarkets or public transportation.

**Figure 1:**
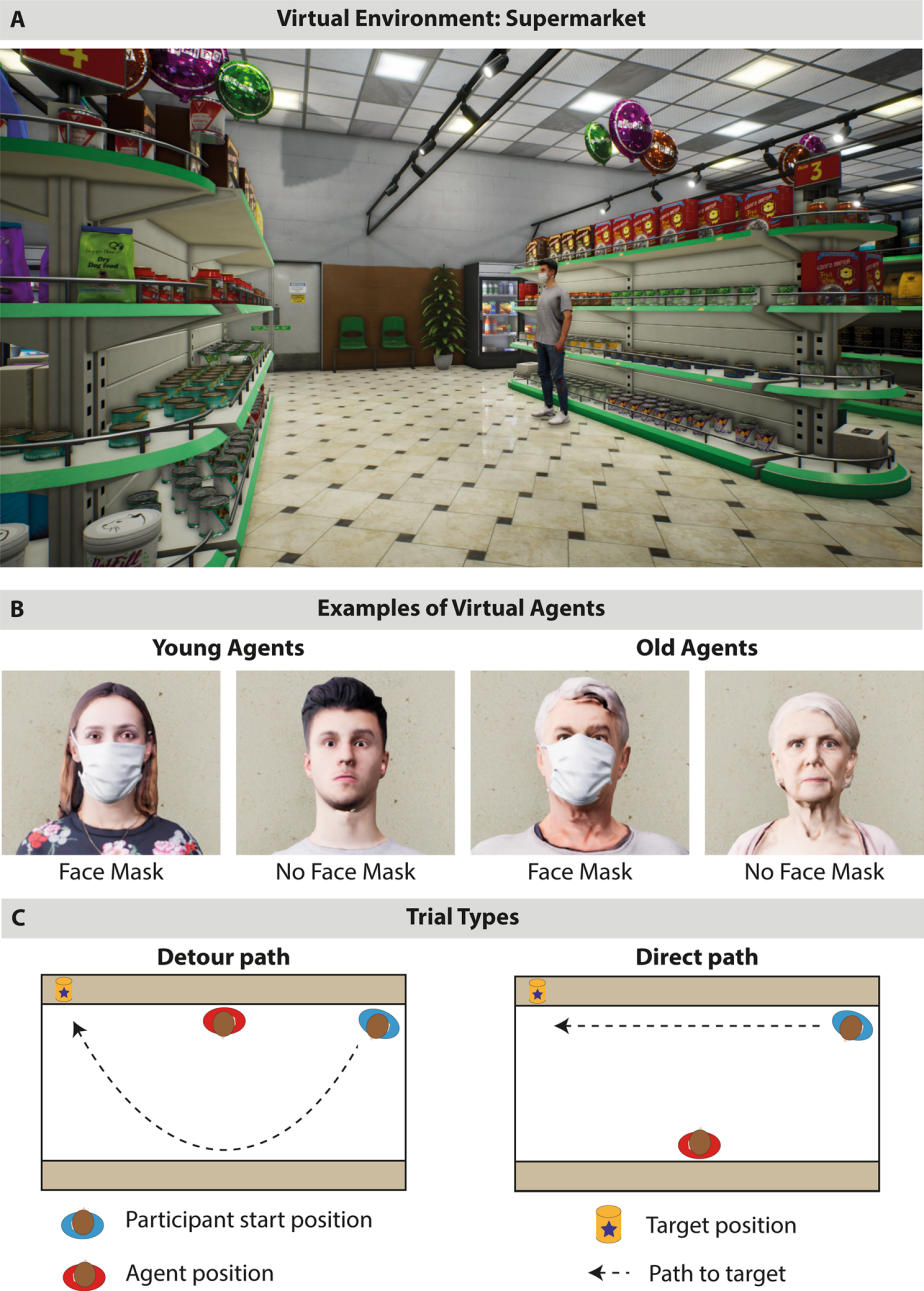
Virtual environment, virtual agents, and trial types. (**A**) Illustration of the virtual supermarket aisle in which the experiment took place. Here a young agent is depicted in the Other Face Mask On condition. (**B**) Example stimuli of young and old virtual agents with and without face masks. Note that every agent was presented both with and without face mask across trials. (**C**) Schematic illustration on the supermarket aisle from a bird’s eye view. Left side shows a detour path trial where the participant (blue) has to take a detour (dashed line) around the agent (red) in order to reach the target (yellow can) while maintaining a minimal distance of 1.5 m. Right side shows a direct path trial where the target can be reached directly and without taking a detour while maintaining a minimum distance of 1.5 m.

In the present study we designed a Virtual Reality experiment that allowed to directly measure the minimal interpersonal distance that participants kept to virtual agents in social situations with a high degree of ecological validity and experimental control. Participants were placed in a virtual supermarket aisle and had to pass-by a virtual agent in order to reach a target location (see Fig. 1). We manipulated whether participants wore face masks as a between-subject factor (*face mask group* vs. *no face mask group*). Furthermore, characteristics of the virtual agents were varied as within-subject factors by displaying agents with and without face masks and by varying agent age as a proxy for the risk status related to Covid-19 (*young/low risk* vs. *old/high risk*). In addition, different situational constraints were implemented, depending on whether participants had to actively maintain the minimal distance of 1.5 m by making a detour when walking to the target location (*detour path*) or whether participants could maintain the minimal distance while reaching the target location directly without making a detour (*direct path*). We hypothesized that both face masks of participants and face masks of agents would decrease the minimal interpersonal distance that participants kept to the agents. Furthermore, we expected that participants would keep greater distances to older compared to younger agents and that the effects of face masks should be pronounced when maintaining a distance of 1.5 m required a detour.

## Results

A mixed ANOVA with the within-subject factors *Face Mask Agent*, *Agent Age*, *Trialtype* and the between-subject factor *Face Mask Participant* was conducted. Face masks worn either by participants or by the virtual agents as well as the age of the agents influenced minimal interpersonal distance depending on the trialtype. The mixed ANOVA revealed a significant three-way interaction of *Face Mask Participant* x *Face Mask Agent x Trialtype*, *F*(1, 82) = 7.02, *p* = 0.010, *η*_*p*_^*2*^ = 0.08, and a significant interaction between *Face Mask Agent x Agent Age x Trialtype, F*(1, 82) = 25.12, *p* < 0.001, *η*_*p*_^*2*^ = 0.23 (see Supplementary Material Table S2 for the full ANOVA table). In order to follow-up on these three-way interactions, step-down ANOVAs were performed separately for detour path trials and direct path trials.

### Detour path trials

Detour path trials, where participants had to actively maintain a distance of 1.5 m while passing the virtual agents, were analyzed using a mixed ANOVA with the factors *Face Mask Participant*, *Face Mask Agent* and *Agent Age* (see Table 1 and Fig. S1). The analysis revealed a significant interaction of *Face Mask Participant* x *Face Mask Agent*, *p*(1, 82) = 7.52, *p* = 0.008, *η*_*p*_^*2*^ = 0.08, a significant interaction of *Face Mask Agent* x *Agent Age*, *p*(1, 82) = 6.59, *p* = 0.012, *η*_*p*_^*2*^ = 0.07, as well as significant main effects of *Face Mask Participant*, *F*(1, 82) = 5.35, *p* = 0.023, *η*_*p*_^*2*^ = 0.06, with closer distances to the agent for the face mask compared to the no face mask group, and *Face Mask Agent*, *p*(1, 82) = 18.85, *p* < 0.001, η_p_^2^ = 0.19, with closer distances to agents with face masks than to agents without face masks.

**Table 1:** Mean (M) and standard deviation (SD) of the minimal distance kept towards virtual agents in cm.

Agent Face Mask	No participant face mask				Participant Face Mask			
	Young		Old		Young		Old	
	M	SD	M	SD	M	SD	M	SD
**Detour path**								
No agent face mask	140.20	33.52	142.83	34.04	126.34	19.30	126.26	18.72
Agent face mask	137.25	29.04	133.07	28.66	125.17	18.58	124.65	16.72
**Direct path**								
No agent face mask	197.61	22.22	204.58	19.08	196.10	15.53	199.82	18.31
Agent face mask	191.55	22.01	207.68	17.21	187.75	23.90	201.31	22.71

Data is shown for all conditions depending on the factors Face Mask participant (no mask vs. mask), Face Mask Agent (no mask vs. mask), Agent Age (young vs. old), Trialtype (detour path vs direct path).

**Figure 2:**
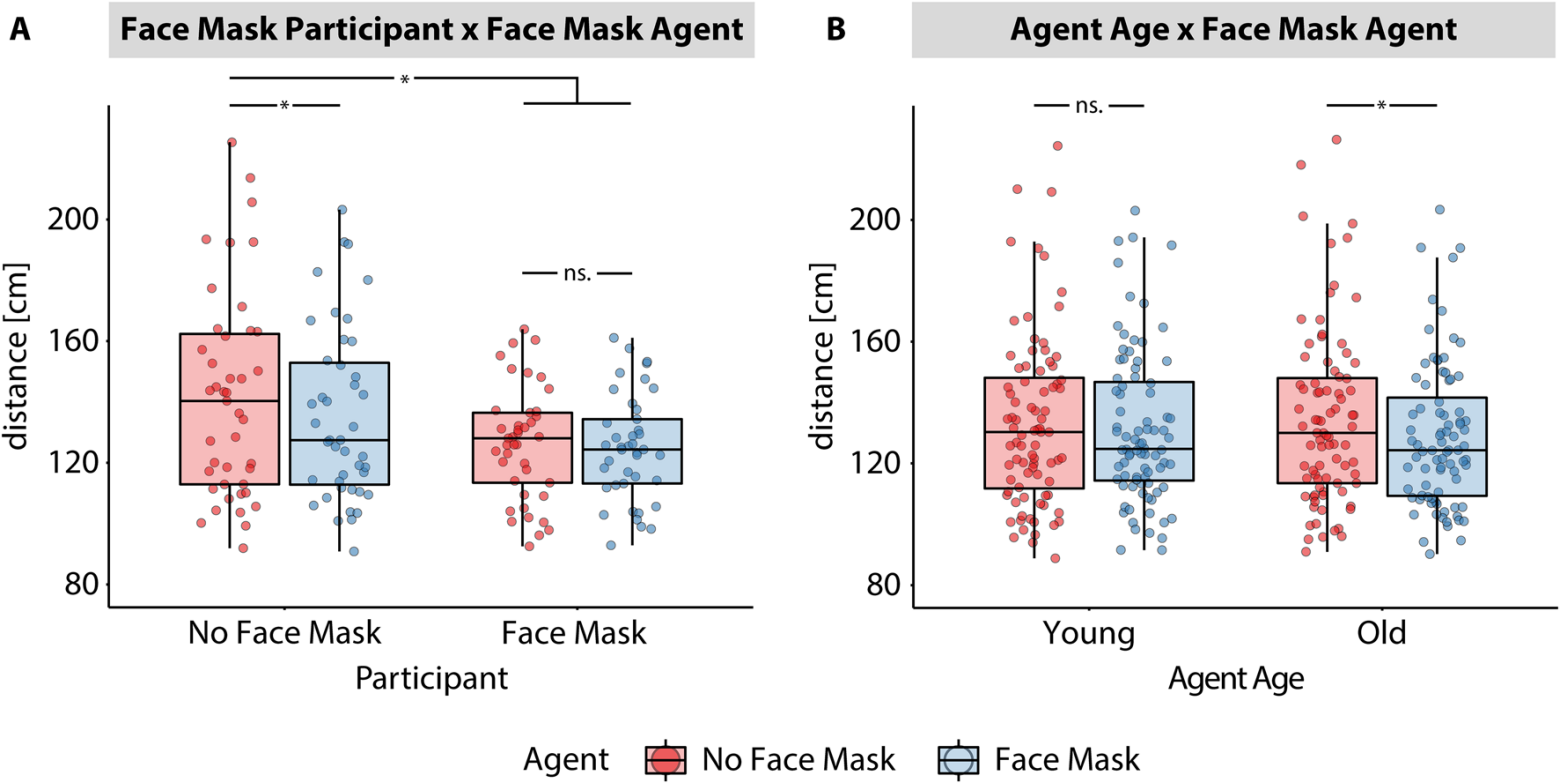
Step down analyses of significant interaction effects in the detour path trials. (**A**) Box plots show minimal distances as a function of “Face Mask Participant” and “Face Mask Agent”. (**B**) Box plots show minimal distances as a function of ”Agent Age” and “Face Mask Agent”.

Post-hoc t-tests were conducted to evaluate the interaction effect between *Face Mask Participant* and *Face Mask Agent* (see Fig. 2A). Participants without face masks kept larger distances to agents without face masks (M = 141.51 cm, SD = 33.61) compared to agents with face masks (M = 135.16, SD = 28.76), t(42) = 4.26, *p* < 0.001, d = 0.65. In contrast, participants with face masks did not differ in the kept distance to agents without face masks (M = 126.30, SD = 18.90) and to agents with face mask (M = 124.91, SD = 17.57), t(40) = 1.393, *p* = 0.192, d = 0.22. In a direct comparison between groups, the no face mask group kept larger distances when confronted to agents without face masks compared to the face mask group when confronted to agents with face masks, t(82) = 2.82, *p* = 0.030, d = 0.62, or without face mask, t(82) = 2.55, *p* = 0.051, d = 0.56 (trend towards significance). The no face mask group, however, when confronted with agents with face masks, did not differ significantly in IPD to the face mask group confronted to agents with and without face masks (all *p* < 0.05).

Post-hoc t-tests were also conducted to follow-up on the interaction effect between *Face Mask Agent* and *Agent Age* (see Fig. 2B). With respect to old agents, there was a significantly reduced distance to agents with face masks (M = 128.96, SD = 23.84) compared to agents without face masks (M = 134.74, SD = 28.72), t(83) = − 5.21, *p* < 0.001, d = 0.57. Whereas, with respect to young agents, there was no significant difference in distance between agents with (M = 131.36, SD = 25.10) and without face masks (M = 133.43, SD = 28.23), t(83) = − 1.63, *p* = 0.107, d = − 0.18.

In summary, both face masks worn by participants and by virtual agents reduced the distance to virtual agents when participants had to actively maintain a distance by taking a detour while passing the agents. The largest distance was kept when both participants and agents did not wear face mask. This distance was reduced when either the agents or the participants wore face masks. When participants wore face masks, there was no additional reduction in distance by face masks of the agents. In addition, with respect to agent age, we observed that participants reduced the distances to old agents without mask in comparison to old agents with mask, while no such effect was observed for young agents.

### Direct path trials

Another mixed ANOVA was conducted for the direct path trials, where the minimum distance of at least 1.5 m could be maintained on the direct path from start to target position (Table 1 and Fig. S2). The analysis revealed an interaction of *Face Mask Agent* x *Agent Age, F*(1, 82) = 19.62, *p* < 0.001, *η*_*p*_^*2*^ = 0.19, as well as a main effect of *Face Mask Agent*, *F*(1, 82) = 6.47, *p* = 0.013, *η*_*p*_^*2*^ = 0.07, with closer distances to agents with face masks compared to agents without face masks, and a main effect of *Agent Age*, *F*(1, 82) = 75.93, *p* < 0.001, *η*_*p*_^*2*^ = 0.48, with closer distances kept to young compared to old agents. There was no significant main effect or interaction including the factor *Face Mask Participant* (all *p* > 0.05).

**Figure 3:**
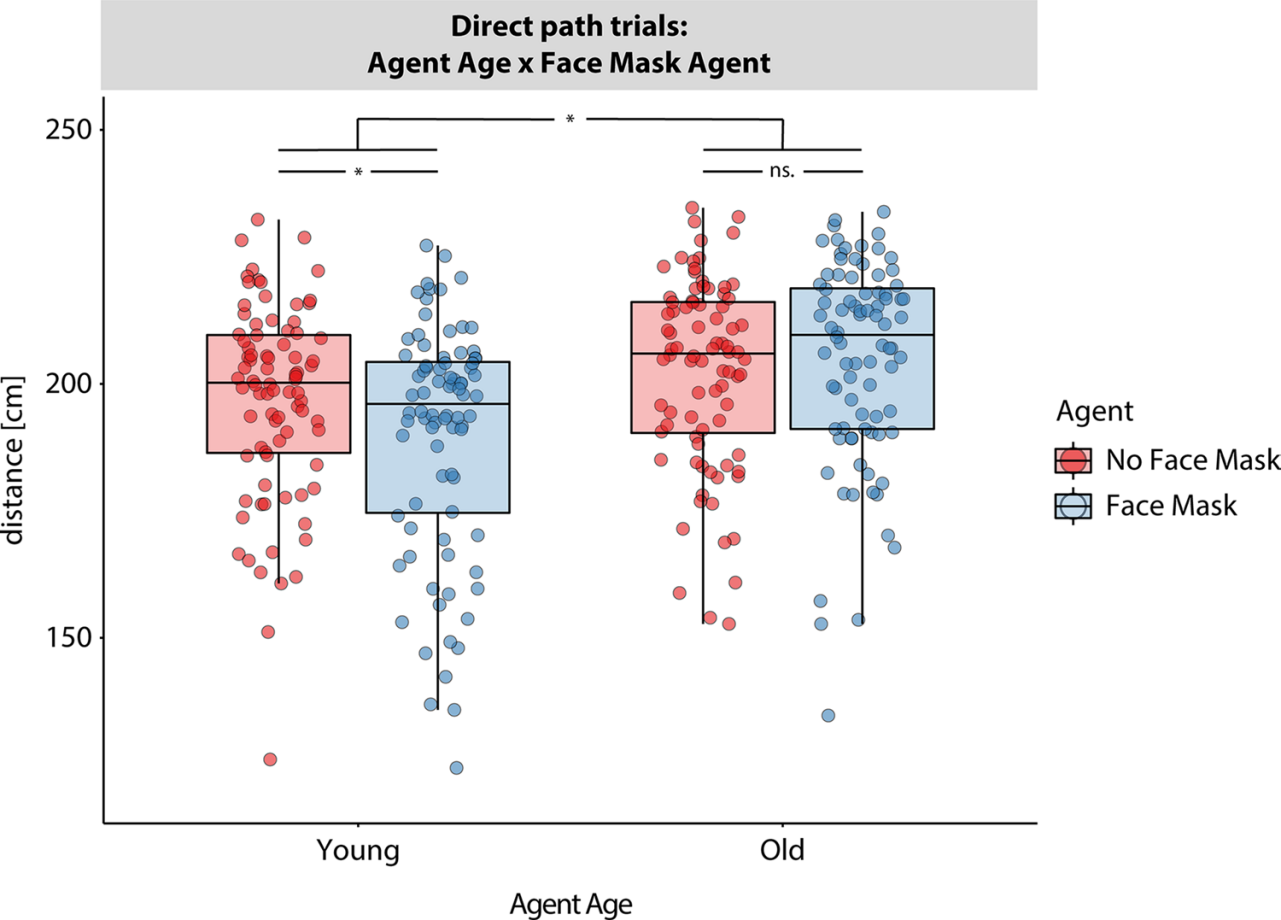
Step down analysis in the direct path trials. Figure shows boxplots overlaid with single subject data points. The within-subject factor “Agent Age” is varied along the x-axis (left for “young agents”, right for “old agents”) and the within-subject factor “Face Mask Agent” is color-coded (“No Face Mask” in red, “Face Mask” in blue). See Supplementary Material for data split according to factor “Face Masks Participant”.

Post-hoc tests (see Fig. 3) showed that distances kept to young agents were significantly closer for agents with face masks (M = 189.69, SD = 22.89) compared to agents without masks (M = 196.87, SD = 19.14), t(83) = − 5.60, *p* < 0.001, d = − 0.61. But there was no significant difference between distances kept to old agents with (M = 205.57, SD = 20.22) and without masks (M = 202.26, SD = 18.75), t(83) = 1.47, *p* = 0.144, d = 0.16. In general, participants kept greater distances to old compared to young agents, irrespective of whether agents wore face masks or not (all *p* < 0.001).

In summary, even when a minimal distance could be maintained without further effort, minimal interpersonal distance was influenced by the age and face masks of the agents. Participants increased distance towards old agents in comparison to young agents. In addition, face masks reduced distances only towards young but not old agents.

### Exploratory analysis

A post-hoc exploratory analysis was conducted using linear mixed effect models in order to test whether individual differences in anxiety, personality, and presence influence minimal interpersonal distance or modulate the effects of face masks (worn by virtual agents or participants) in the detour path condition. There were no significant interaction effects for any individual measure with the factors *Face Mask Agent* and *Face Mask Participant* (all *p* > 0.1). Models revealed significant main effects of physical presence (β = 12.43, SE = 3.94, t(78.73) = 3.15, *p* = 0.002) and social presence (β = 10.07, SE = 3.68, t(80.57) = 3.15, *p* = 0.008) of the multimodal presence scale. There were no significant main effects for any other individual measure (all *p* > 0.1*)*. Participants who reported higher physical and social presence kept a greater minimal distance towards virtual agents but this effect was not modulated by face masks. See Supplementary Material for graphs depicting the linear effect of presence (Figs. S3 and S4) and all model summaries (Tables S3–S10).

## Discussion

Wearing face masks reduced participant’s minimal interpersonal distance towards virtual agents in a Virtual Reality paradigm. In line with our hypotheses, we found that face masks worn by the participants or by the agents reduced IPD between participants and virtual agents. The observed interaction between the factors agent face mask and participant face mask showed that face masks of the virtual agents reduced minimal distance only when participants did not wear face masks, while there was no effect of agent face mask when participants did wear face masks. Importantly, participant face mask effects were only observed when keeping a minimum distance of 1.5 m was effortful but not when this distance could be maintained without further effort. In addition, the effect of agent age on distance behavior also varied as a function of effort related to maintaining a 1.5 m distance. In the direct path condition, where no additional effort was required, participants were sensitive to the age of agents and kept greater distances to older compared to younger agents. Furthermore, face masks worn by the agents only lead to a reduction of distances towards young but not old agents. In contrary, in the detour path condition, there was no overall effect of agent age, instead agent face masks even reduced the distance to older agents. Finally, an exploratory analysis could show that increased presence in virtual reality was associated with an increased minimal distance towards virtual agents. In summary, our data show that face masks result in reduced minimal interpersonal distance. Importantly, these effects are modulated by situational constraints which relate to the effort of keeping a safe distance.

In line with risk compensation theories^5,8^, wearing face masks as a safety behavior lead to a reduction of physical distancing, a behavior that is associated with an increased risk for contracting Covid-19^3,25^. We found an interaction effect showing that face masks of other persons only resulted in a distance reduction when participants did not wear face masks by themselves. Thereby, our results extend previous findings of online experiments that showed reduced distance preferences due to face masks^21–26^. Given that successful airborne virus containment relies on the combination of face masks and distancing measures^2^, our findings have important implications on safety behavior policies and demonstrate the need to communicate the importance of keeping a physical distancing despite mask wearing.

The present experimental design allowed to investigate the role of situational constraints on risk compensation behavior. Risk compensation was most pronounced when the recommended distance of 1.5 m had to be maintained by making a detour but was less prominent when the minimum recommended distance could be maintained without additional effort. Thus, risk compensation behavior seems to be increased when adherence to safety measures is costly, whereas compensatory behavior is less prominent when adherence to safety measures can be achieved without additional effort. Importantly, the detour path condition induced a conflict between the goal to reach the target item on a direct path and the recommendation to keep a minimal distance to other persons, which had to be resolved using self-control^27^, while the direct path conditions didn’t induce such a conflict. Consequently, one might speculate that reduced self-regulatory capacity might account for reduced rule adherence in the detour compared to the direct path conditions^28^. Situational constraints might further explain conflicting results from previous studies that did not observe reductions in distances due to face masks^29^. Taken together, the findings demonstrate a modulatory influence of extrinsic situational constraints related to the effects of face masks on physical distancing. This has important implications, given that there are numerous everyday situations where adherence to safety measures is effortful (e.g. in public transportation or when in a hurry).

In addition to situational constraints, participant were sensitive towards the age of the virtual agents. High age has been described as a risk factor for severe health implications resulting from an infection with Covid-19^30^. In the present study, participants were aware of this associations and kept greater distances to old agents than to young agents. Crucially, this effect was only observed in the direct path condition but not in the detour path condition. Instead, in the detour conditions participants even reduced IPD towards elderly with face masks. Apparently, participants knew about the risk status of elderly^31^ but had difficulties in adapting their behavior depending on the situational constraints. As elderly also showed difficulties in actively establishing a safe distance to an approaching stranger in a previous study^18^, the present results need to be taken into account when safety policies for risk groups are discussed.

The present study used a virtual reality paradigm with high experimental control to investigate distance behavior as a function of face masks. Furthermore, using VR allowed the unobtrusive measurement of real distance behavior. However, while VR has been successfully used to study IPD in previous studies^14,32^, there are limitations that need to be discussed. First, there was no real risk of infection at any point during the experiment. While this is a strength from an ethical point of view, it remains possible that participants’ behavior changed because participants knew that neither they nor the agents were at a risk of infection. Note, however, that in this case face masks or agent age should have no effect on IPD. In contrast, our findings show that these factors influenced behavior despite being task irrelevant. As a further limitation, the present study might differ from a real-world setting as there were no social consequences from the virtual agents. Furthermore, previous studies showed differences in social perception and social judgments between virtual characters and real persons^33,34^. Therefore, motives related to politeness or other social context factors might have been less impactful. In line with this argument, we found that participants with an increased physical presence (the sense of being in the virtual room) or social presence (the sense of being there with another person) kept greater distance towards virtual agents. Differences with respect to presence and social perception might also be the reason for the mixed findings between studies using computer-paradigms^20,23^ and studies using observations in real-life situations^29^. While we did not find that presence modulated the effects of face masks in the current study, future research should explicitly manipulate presence or participants’ beliefs about agency of virtual agents in order to test whether social context affects the influence of face masks on IPD.

Finally, it should be acknowledged that the virtual agents in the current study were presented with surgical face masks (in the face mask conditions). Because surgical face masks compared to N95/FFP2 masks have been shown to be less effective in reducing viral transmission^2^ and in wearer protection^35^, mask type might also play an important role with respect to risk compensation behavior^36^. Based on the present data one could expect increased risk compensation for N95 masks compared to surgical masks. This hypothesis should be tested in futures studies. Please also note that the mask mandate that was active during the time of data acquisition did not specify masks types and therefore surgical masks were common.

In conclusion, using an ecologically valid Virtual Reality paradigm, the present study provides experimental evidence that wearing face masks reduces the minimal interpersonal distance kept to virtual strangers. This effect was observed both for face masks worn by the participants and face masks worn by the agents. In addition, the present findings suggest that situational constraints influence to which degree face masks result in risk compensation behavior. These findings have important implications both for safety policies as well as for future studies investigating risk compensation behavior.

## Methods

### Participants

Eighty-six healthy students were recruited at the University of Regensburg. Participants did not report mental or neurological disorders. Due to technical problems during data acquisition, two participants had to be excluded from data analysis. The final sample included N = 84 participants (M_Age_ = 21.79, SD_Age_ = 3.64, 76 female). Participants were randomly allocated to the groups *face mask* (N = 41) and *no face mask* (N = 43). According to a power analysis, the present study allowed to detect medium effect sizes of d = 0.63 for between subject comparisons and small effect sizes of d = 0.31 for within-subject comparisons (both with 1-β = 0.80 and α = 0.05, independent and paired t-tests respectively). Experimental procedures were in line with the Declaration of Helsinki and approved by the ethics committee of Regensburg University. The study was conducted according to the approved guidelines. All participants gave written informed consent.

Data acquisition was conducted from 17th of August to 1st of December 2020 in Regensburg, Germany. During this time a mask mandate was active in shops, restaurants, and public transportation. Different types of face masks (cloth, surgical, N95) were accepted in this mask mandate. According to the Robert Koch Institute^37^ incidences of COVID-19 infections in Germany rose from 8.5 cases/100.000 inhabitants (17th August 2020) to 136.6 cases/100.000 (1st December 2020). During the time of testing there were no COVID-19 vaccinations available in Germany.

### Questionnaires

Questionnaires before and after the experiment were used to assess demographic information, general anxiety (*State Trait Anxiety Inventory*^38^), social anxiety (*Social Phobia Inventory*^39,40^), sensitivity to reward and punishment^41^, hypochondria (*Whiteley-Index*^42^)*,* as well as cyber sickness (*Simulator Sickness Questionnaire*^43^)*,* and presence (*Multimodal Presence Scale*^44^*, iGroup Presence Questionnaire*^45^). We conducted Welch two-sample t-tests in order to investigate whether the two groups *face mask* and *no face mask* differed in any of the assessed questionnaires. Group means and results of the statistical tests are reported in the Supplementary Material. There were no significant group differences.

### Experimental paradigm

The experiment was conducted within a Cave Automatic Virtual environment (CAVE) system with a size of 3.6 m × 2.4 m × 2.5 m. Participants wore shutter glasses with attached motion tracker targets (Advance Realtime Tracking GmbH) and VR was projected on the four surrounding walls and the floor of the CAVE (Barco F50 WQX6A projectors with a resolution of 2560 by 1600 pixels). An additional motion tracker target was attached to the dominant hand of the participants. VR was rendered using Unreal Engine 4 (v 4.23, Epic Games Inc.) in a cluster of ten computers (i7-4790 k, GeForce 1080, 16 GB RAM). Sounds were presented via a surround sound system (Yamaha HTR-3066). Using a CAVE set-up allowed participants to move inside the virtual environment by themselves without using an avatar. Consequently, participants in the face mask condition were actually wearing a real face mask.

Virtual environment (see Fig. 1A) resembled a supermarket and was created using a commercial asset pack from the Unreal 4 marketplace (https://www.unrealengine.com/marketplace/en-US/product/supermarket). In the virtual scenario a typical supermarket aisle was centered within the CAVE so that the shelves were aligned parallel to the long walls of the CAVE and the space between walls reflected the aisle. In the shelves typical supermarket products were presented like cans or cereal boxes. There were no shelves on the short CAVE walls, allowing virtual agents to enter and leave the aisle. Participants could move freely within the aisle.

Eight different virtual agents were taken from the Renderpeople library (https://renderpeople.com/). Agents consisted of four younger adults and four elderly (two male and two female per age group). For every agent, a version with and without a face mask was created (see examples in Fig. 1B). The masks resembled surgical face masks. All agents were animated to walk and to show idle movements when standing (breathing and slight movements of the upper body).

### Procedure

Participants were screened for Covid-19 symptoms, received written experimental instructions and filled in questionnaires regarding demographic information, anxiety, and personality. Due to a mask mandate inside the university buildings all participants arrived wearing a face mask. Next, participants put on the VR shutter glasses and a motion tracking target was attached to the back of the dominant hand. Participants were instructed that they had the task to collect items in a virtual supermarket and that there would be other virtual agents. There were no instructions about keeping distance and face masks of the agents (see supplementary material for the full task instructions). In the no face mask group, participants were asked to remove their own face masks before the experiment, in the face masks group, participants left their face masks on and no further instruction was given. After this, participants entered the CAVE, where the supermarket environment was already presented on walls and floor. The CAVE door was closed once the participants were inside, so that the virtual environment was presented in a 360° view and allowed free movement of the participant.

In total, 80 trials were presented over the course of the experiment. Trial order was pseudo-randomized with respect to experimental conditions, with no more than three repetitions of the same Face Mask Agent, Agent Age or Trial Type condition. Every trial started with the visual presentation of “footsteps” on the floor in one of the four corners of the CAVE which served as visual markers for participants’ start position in each trial. Participants were instructed to stand on top of these footsteps and to face towards the aisle at trial begin. Next, a virtual agent entered the aisle from the opposite end relative to the participant, so that the distance of at least 1.5 m was maintained between participant and agent. The agent moved to one of six position in the aisle (three positions in front of each shelve) and then turned to face towards the aisle. In every trial the agent’s position was at least 1.5 m away from the start position of the participant. Once the agent had taken a position, a target item in one of the shelves lit up in red color. The marked target items were always in one of the corners of the CAVE, but never in the corner of the start position. Next, an auditory cue informed participants to perform the task, i.e. to “collect” the marked item. In order to “collect” an item, participants had to move towards the item and, once they had reached the correct position in front of the shelve, move their dominant hand to the height of the item. Once participants hand reached the height of the item, the color of the item turned to green and another auditory cue was presented. The visual and auditory cue informed the participant that the item had been successfully “collected”. After this, the agent left the aisle (again towards the opposite end relative to the participant) and the next trial started. The end position in one trial always served as the start position of the next trial. Participants were instructed to turn towards the aisle once they had “collected” the item.

Importantly, by varying the start position of the participant as well as the position of the virtual agent and the position of the target we created different trial types. These trials differed with respect to whether the minimum distance of 1.5 m between participant and virtual agent could be maintained on the direct path from the start position to the target position (direct path trials) or whether participants had to deviate from the direct path and make a detour in order to maintain the minimum distance of 1.5 m (detour path trials). See Fig. 1C for example configurations relating to detour path trial (left) and direct path trial (right). The experiment contained 48 detour path trials and 32 direct path trials. The conditions face mask agent and agent age were equally distributed within trial types (i.e. 12 trials per condition in the detour path trials and 8 trials per condition in the direct path trials).

### Data recording, data processing and statistics

Participants’ head position in xyz-coordinates was continuously recorded with a sample rate of 45 Hz using the motion tracking targets attached to the shutter glasses. In addition, the xyz-coordinates of the virtual agents were recorded with the same sample rate once the agents entered the supermarket aisle. For data analysis we calculated the Euclidian distance between participant position and agent position in the xy-plane using custom scripts in Matlab 8.1 (Mathwork, Natik, MA). In a next step, the minimal distance between participant and agent was determined for every trial. Data points were excluded from analysis when participants were closer than 80 cm to the CAVE walls in order to restrict the influence of the physical barriers on participants’ movements.

Finally, minimal distances were averaged across trials into conditions according to *Trialtype* (detour path trials, direct path trials), *Face Mask Agent* (face mask, no face mask) and *Agent Age* (young, old) for every participant and exported for statistical analyses. Statistical analysis was conducted in the R environment^46^. We calculated a mixed-effect ANOVA including the within-subject factors *Trialtype*, *Face Mask Agent* and *Agent Age* and the between-subject factor *Face Mask Participant.* Post-hoc t-tests were conducted to follow-up on significant interactions and corrected for multiple testing according to Holm^47^. For all analyses the significance level was set to α = 0.05.

An exploratory post-hoc analysis was conducted to investigate whether individual differences in anxiety and presence measures modulate effects of face masks (participant and agent face mask) on minimal interpersonal distance in the detour condition. For that reason linear mixed effect models were calculated using the *lme4* package^48^. Fixed effects were defined as main effects and interaction effect of *Face Mask Agent* (sum coded: no face mask = − 1, face mask = 1) and *Face Mask Participant* (sum coded: no face mask = − 1, face mask = 1)*,* the main effect of the individual measure as continuous predictor, as well as interactions of the individual measure with *Face Mask Agent* and *Face Mask Participant.* For all models a maximal random effect structure was implemented by including random slopes for *Face Mask Agent* and random intercepts per subjects. Models were fit by restricted maximum likelihood. Separate models were calculated to investigate individual predictors of state/trait anxiety^38^, social phobia^39^, hypochondria^42^, sensitivity to reward/punishment^41^, as well as physical and social presence^44^. Parameter estimates were evaluated using t-tests with Satterthwaite approximations of degrees of freedom^49^. Full model summaries are presented in the supplementary material (Tables S3–S10).

### Ethics declaration

The study was reviewed and approved by Ethics Committee of the University of Regensburg. Experimental procedure were in line with the Declaration of Helsiniki. The participants provided their written informed consent to participate in this study. The study was conducted according to the approved procedure.

## Supplementary Information

Supplementary Information.

## Data Availability

Anonymized data as well as all scripts related to data processing and analysis are available in a public repository (https://osf.io/htrx3/).
