# Supplementary material for: Face masks reduce interpersonal distance in virtual reality
Source: Sci Rep. 2022 Feb 9;12:2213. doi: 10.1038/s41598-022-06086-x (PMC8828850; doi:10.1038/s41598-022-06086-x)
Supplement: Supplementary file 1 — Supplementary Information. [file 41598_2022_6086_MOESM1_ESM.pdf]

Supplementary Material: ***Face masks reduce interpersonal distance in virtual reality***

Leon O. H. Kroczek*[1], Stephanie Böhme[2] & Andreas Mühlberger[1]

[1] Department of Psychology, Clinical Psychology and Psychotherapy, Universität Regensburg

[2] Department of Psychology, Clinical Psychology and Psychotherapy, Friedrich-Alexander-Universität Erlangen-Nuremberg

Experimental task instructions

Here we present a literal translation of the task instructions that were given to the participants before the start of the experiment:

“Study: The influence of the Covid-19 pandemic on behavior in everyday situations

Thank you for participating in this study. The goal of the present study is to investigate how everyday behavior, like going shopping in a supermarket is influenced by the present Covid-19 situation.

Task:

You will be placed in a virtual supermarket together with other virtual persons. Your task is to collect different items. Once, a particular item “lights” up in blue color, please move to the item and collect it by moving your dominant hand towards the item.”

Comparisons between “face mask” and “no face mask” groups

Table S1: Mean and standard deviations for age and questionnaires separately for the “face mask” and “no face mask” group. Group comparisons were performed using Welch two sample t-tests.

**Measure**	**Face Mask**<br>Mean (sd)	**No Face Mask**<br>Mean (sd)	**t-test**<br>p-value
Age	22.24 (4.64)	21.16 (2.26)	.183
SPIN	18.59 (8.60)	16.70 (8.74)	.321
STAI Trait	38 (10.17)	36.70 (7.52)	.508
STAI State Pre	35.41 (8.10)	34.93 (8.13)	.785
STAI State Post	36.61 (9.76)	35.63 (8.88)	.632
Whiteley-Index	1.85 (1.85)	2.09 (1.69)	.538
SR	9.6 (3.54)	9.72 (4.27)	.888
SP	10.65 (4,11)	10.29 (4.70)	.650
SSQ	6.83 (5.51)	5.42 (4.35)	.198
MPS-Physical	2.67 (0.65)	2.73 (0.70)	.689
MPS-Social	2.15 (0.67)	2.25 (0.81)	.540
IPQ-G	3.53 (1.54)	3.90 (1.34)	.237
IPQ-SP	4.17 (0.84)	4.12 (1.07)	.847
IPQ-INV	3.00 (1.40)	3.18 (1.21)	.539
IPQ-ER	2.22 (1.17)	2.35 (1.03)	.571

Abbreviations: SPIN = Social Phobia Inventory, STAI = State Trait Anxiety Inventory, SR = Sensitivity to Reward, SP = Sensitivity to Punishment, SSQ = Simulator Sickness Questionnaire, MPS = Multimodal Presence Scale, IPQ = iGroup Presence Questionnaire with subscales G = General, SP = Spatial Presence, INV = Involvement and ER = Experienced Realism

Detour path trials

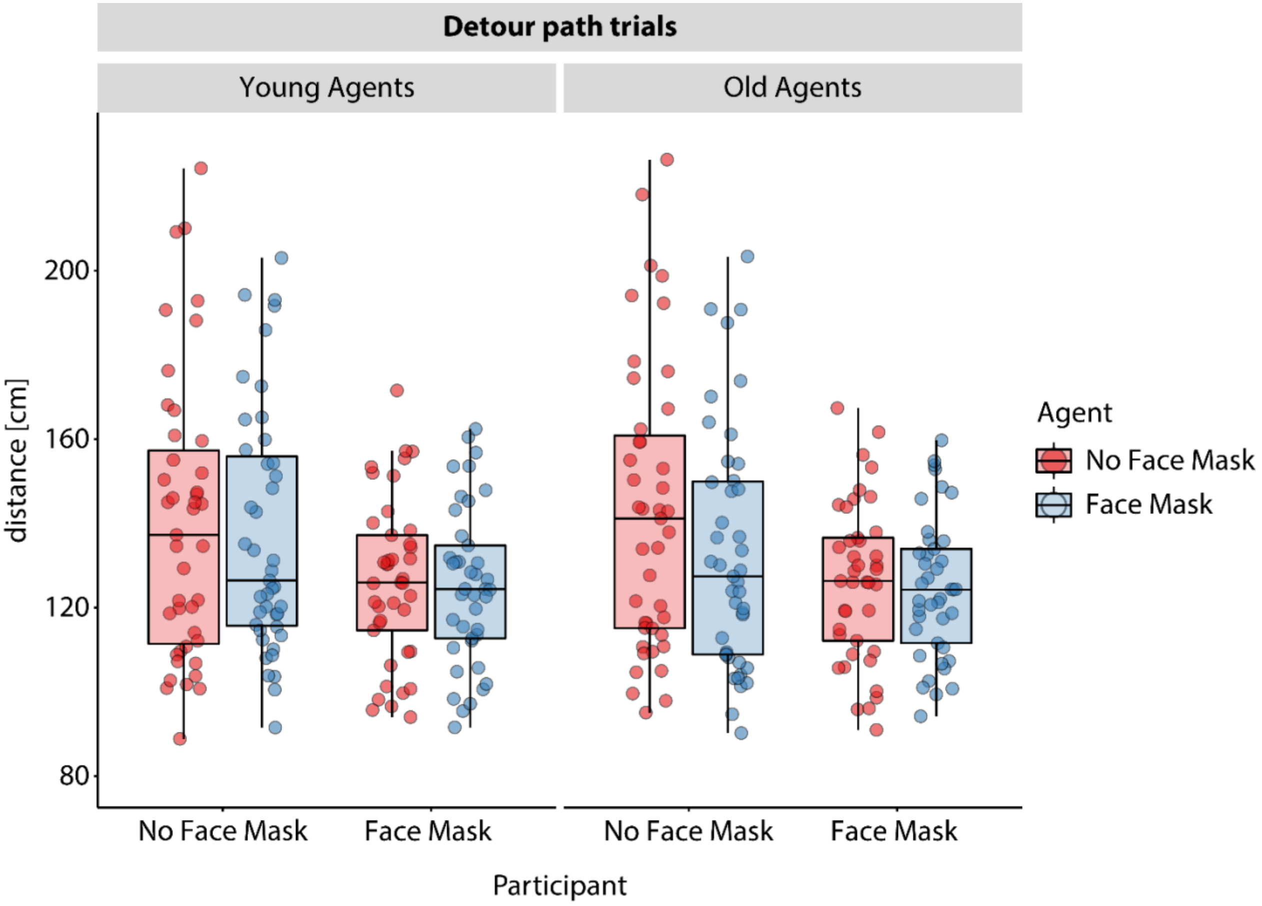

Figure S1: Minimal distances between participants and agents in the detour path trials. *Figure shows boxplots of single conditions overlaid with single subject data points. The between-subject factor "Face Mask Participant" is varied along the x-axis, the within-subject factor "Face Mask Agent" is color-coded ("No Face Mask" in red, "Face Mask" in blue) and the within-subject factor "Agent Age" is varied between graphs (left for "young agents", right for "old agents").*

Direct path trials

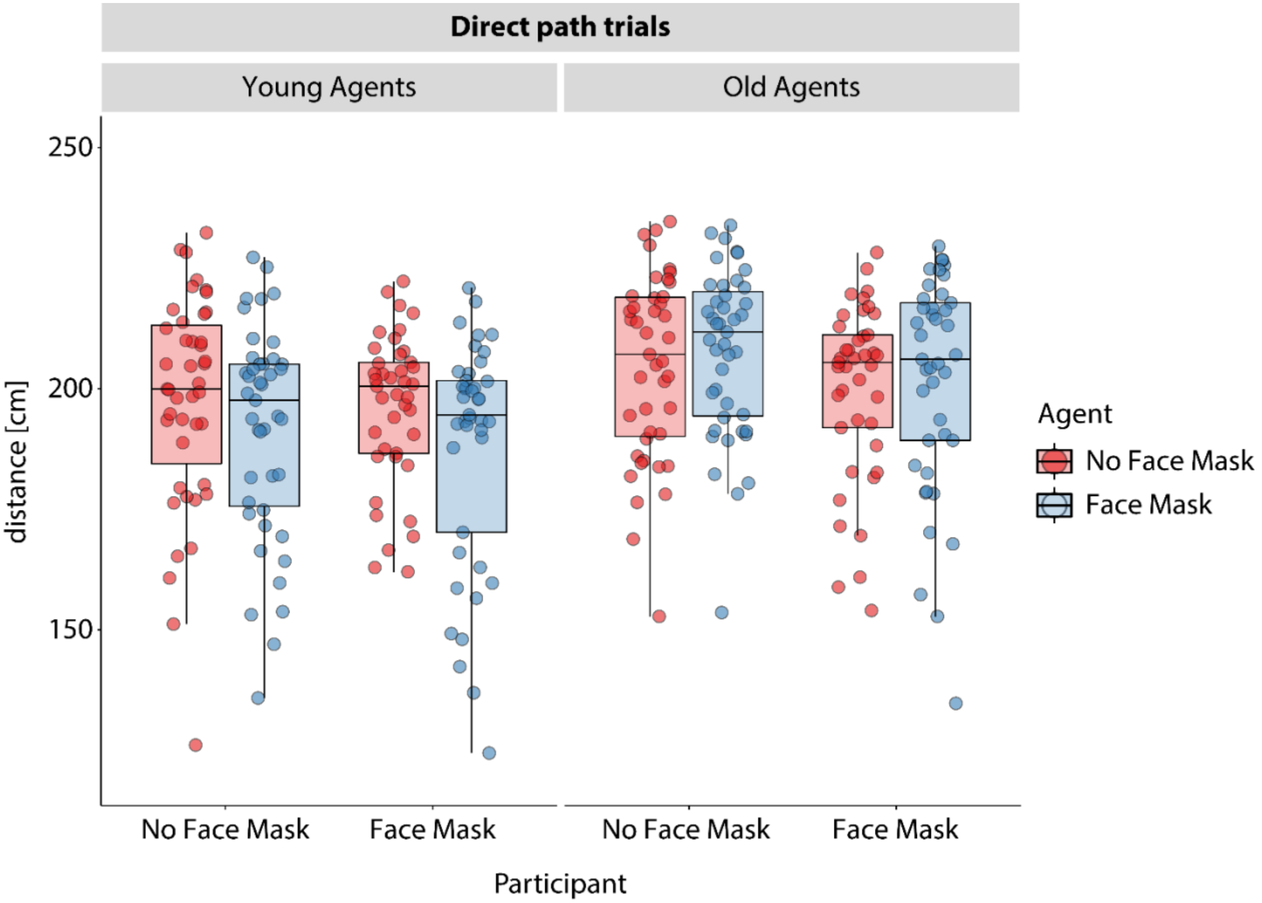

Figure S2: Minimal distances between participants and agents in the direct path trials. *Figure shows boxplots of single conditions overlaid with single subject data points. The between-subject factor "Face Mask Participant" is varied along the x-axis, the within-subject factor "Face Mask Agent" is color-coded ("No Face Mask" in red, "Face Mask" in blue) and the within-subject factor "Agent Age" is varied between graphs (left for "young agents", right for "old agents").*

Full ANOVA results of mixed ANOVA: Face Mask Participant x Face Mask Agent x Agent Age x Trialtype

Table S2: Results of the mixed ANOVA with the within-subject factors Face Mask Agent, Agent Age, Trialtype and the between-subject factor Face Mask Participant

**Effect**	df_{Num}	df_{Den}	F	p	η_p^2
Face Mask Participant	1	82	4.23	.043	.05
Face Mask Agent	1	82	22.91	< .001	.22
Agent Age	1	82	64.26	< .001	.44
Trialtype	1	82	674.39	< .001	.89
Face Mask Participant x Face Mask Agent	1	82	1.28	.261	.02
Face Mask Participant x Agent Age	1	82	1.04	.311	.01
Face Mask Participant x Trialtype	1	82	2.86	.095	.03
Face Mask Agent x Agent Age	1	82	5.22	.025	.06
Face Mask Agent x Trialtype	1	82	1.31	.255	.02
Agent Age x Trialtype	1	82	64.43	< .001	.44
Face Mask Participant x Face Mask Agent x Agent Age	1	82	1.94	.167	.02
Face Mask Participant x Face Mask Agent x Trialtype	1	82	7.02	.010	.08
Face Mask Participant x Agent Age x Trialtype	1	82	1.63	.206	.02
Face Mask Agent x Agent Age x Trialtype	1	82	25.12	< .001	.23
Face Mask Participant x Face Mask Agent x Agent Age x Trialtype	1	82	1.16	.285	.01

Note. df_{Num} indicates degrees of freedom numerator. df_{Den} indicates degrees of freedom denominator. η_p^2 indicates partial eta-squared.

Exploratory analysis: Effect of presence on minimal IPD

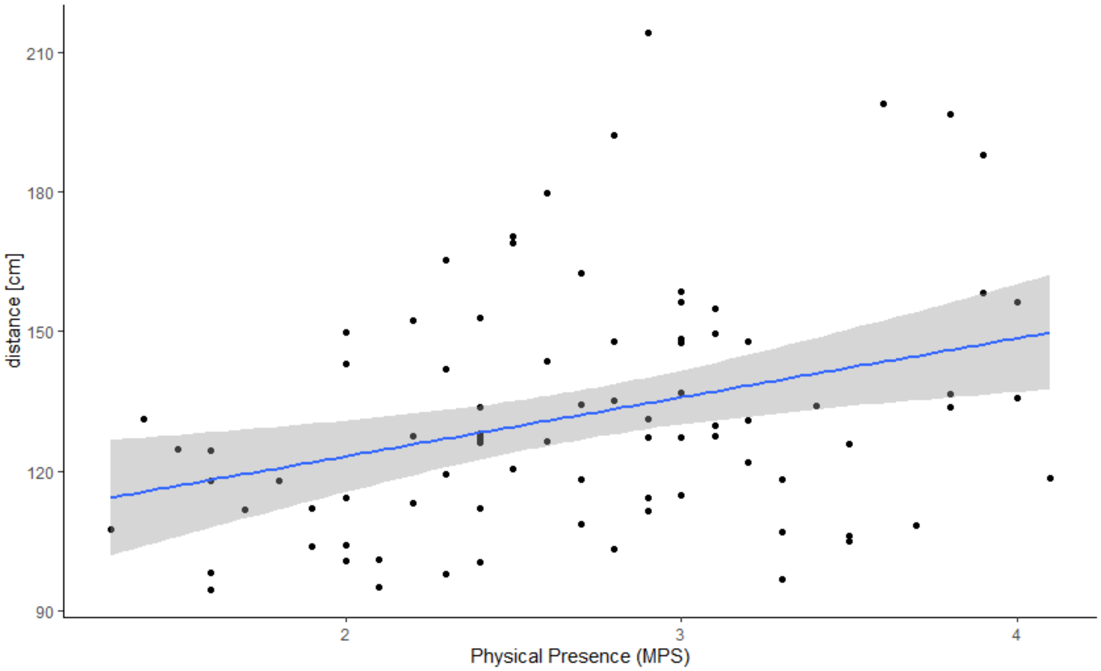

Figure S3: Effect of physical presence on minimal interpersonal distance. *Individual scores in the physical presence subscale of the Multimodal Presence Scale are plotted on the x-axis. Y-axis shows distance values relating to the minimal distance that participants kept between themselves and the virtual agents. Blue line shows linear model fit. Shaded area reflects SE of model fit.*

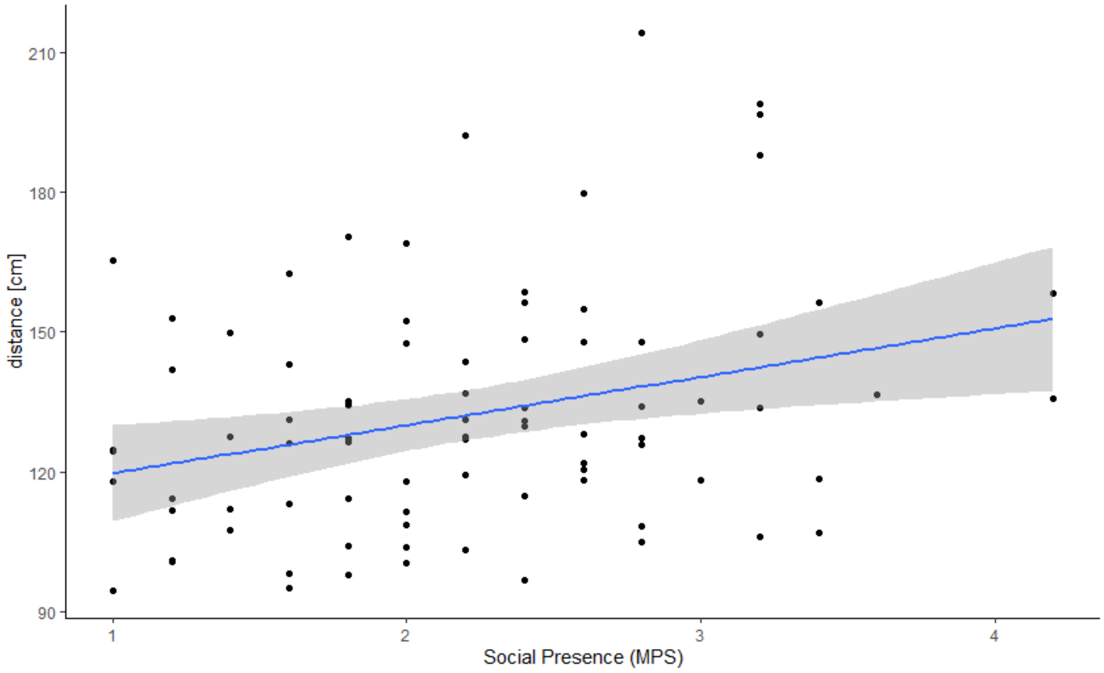

Figure S4: Effect of social presence on minimal interpersonal distance. *Individual scores in the social presence subscale of the Multimodal Presence Scale are plotted on the x-axis. Y-axis shows distance values relating to the minimal distance that participants kept between themselves and the virtual agents. Blue line shows linear model fit. Shaded area reflects SE of model fit.*

Exploratory analysis: Model summaries

Physical Presence (Multimodal Presence scale)

Table S3. Linear mixed effect model including the individual measure of physical presence (MPS) for the analysis of minimal distance in the detour path trials. Model equation: minimal distance ~ 1 + Face Mask Agent Face Mask Participant + Face Mask Agent* physical presence + Face Mask Participant* physical presence + (1+ Face Mask Agent| Subject)*

Predictors	*Estimates*	*SE*	*95% CI*	*t*	*df*	*p*
Intercept	98.41	10.96	76.59 – 120.23	8.98	79.66	**<0.001**
Face Mask Agent	0.05	1.88	-3.68 – 3.79	0.03	81.00	0.977
Face Mask Participant	-9.25	9.90	-28.94 – 10.44	-0.93	82.13	0.353
Physical Presence	12.43	3.94	4.59 – 20.27	3.15	79.73	**0.002**
Face Mask Agent x Face Mask Participant	1.22	0.45	0.32 – 2.12	2.69	81.00	**0.009**
Face Mask Agent x Physical Presence	-0.74	0.67	-2.08 – 0.60	-1.09	81.00	0.278
Face Mask Participant x Physical Presence	1.20	3.53	-5.83 – 8.24	0.34	80.00	0.734

Social Presence (Multimodal Presence scale)

Table S4. Linear mixed effect model including the individual measure of social presence (MPS) for the analysis of minimal distance in the detour path trials. Model equation: minimal distance ~ 1 + Face Mask Agent Face Mask Participant + Face Mask Agent* social presence + Face Mask Participant* social presence + (1+ Face Mask Agent| Subject)*

Predictors	*Estimates*	*SE*	*95% CI*	*t*	*df*	*p*
Intercept	109.87	8.50	92.96 – 126.78	12.93	80.27	**<0.001**
Face Mask Agent	-0.22	1.42	-3.04 – 2.60	-0.15	81.00	0.878
Face Mask Participant	-8.35	7.75	-23.76 – 7.06	-1.08	83.36	0.284
Social Presence	10.07	3.68	2.75 – 17.39	2.74	80.57	**0.008**
Face Mask Agent x Face Mask Participant	1.20	0.45	0.30 – 2.10	2.66	81.00	**0.009**
Face Mask Agent x Social Presence	-0.78	0.61	-1.99 – 0.43	-1.28	81.00	0.205
Face Mask Participant x Social Presence	1.13	3.32	-5.48 – 7.73	0.34	80.00	0.735

Trait Anxiety (STAI Trait)

Table S5. Linear mixed effect model including the individual measure of trait anxiety (STAI) for the analysis of minimal distance in the detour path trials. Model equation: minimal distance ~ 1 + Face Mask Agent Face Mask Participant + Face Mask Agent*STAI_Trait + Face Mask Participant* STAI_Trait + (1+ Face Mask Agent| Subject)*

Predictors	*Estimates*	*SE*	*95% CI*	*t*	*df*	*p*
Intercept	118.93	12.35	94.37 – 143.50	9.63	81.85	**<0.001**
Face Mask Agent	-0.10	1.97	-4.03 – 3.82	-0.05	81.00	0.958
Face Mask Participant	-7.59	11.20	-29.87 – 14.69	-0.68	81.86	0.500
Trait Anxiety	0.35	0.32	-0.29 – 0.99	1.08	82.13	0.284
Face Mask Agent x Face Mask Participant	1.27	0.45	0.37 – 2.18	2.80	81.00	**0.006**
Face Mask Agent x Trait Anxiety	-0.05	0.05	-0.15 – 0.05	-0.95	81.00	0.343
Face Mask Participant x Trait Anxiety	0.03	0.29	-0.55 – 0.61	0.09	80.00	0.927

State Anxiety (STAI State)

Table S6. Linear mixed effect model including the individual measure of state anxiety (STAI) for the analysis of minimal distance in the detour path trials. Model equation: minimal distance ~ 1 + Face Mask Agent Face Mask Participant + Face Mask Agent*STAI_State + Face Mask Participant* STAI_State + (1+ Face Mask Agent| Subject)*

Predictors	*Estimates*	*SE*	*95% CI*	*t*	*df*	*p*
Intercept	118.78	12.36	94.18 – 143.38	9.61	80.30	**<0.001**
Face Mask Agent	-2.51	2.05	-6.58 – 1.57	-1.22	81.00	0.225
Face Mask Participant	9.04	10.98	-12.80 – 30.88	0.82	82.15	0.413
State Anxiety	0.38	0.34	-0.30 – 1.06	1.10	80.29	0.273
Face Mask Agent x Face Mask Participant	1.24	0.46	0.33 – 2.14	2.72	81.00	**0.008**
Face Mask Agent x State Anxiety	0.02	0.06	-0.10 – 0.13	0.29	81.00	0.776
Face Mask Participant x State Anxiety	-0.44	0.30	-1.04 – 0.16	-1.46	80.00	0.149

Social Anxiety (Social Phobia Inventory, SPIN)

Table S7. Linear mixed effect model including the individual measure of social anxiety (SPIN) for the analysis of minimal distance in the detour path trials. Model equation: minimal distance ~ 1 + Face Mask Agent Face Mask Participant + Face Mask Agent*SPIN + Face Mask Participant* SPIN + (1+ Face Mask Agent| Subject)*

Predictors	*Estimates*	*SE*	*95% CI*	*t*	*df*	*p*
Intercept	122.72	6.30	110.19 – 135.26	19.49	80.07	**<0.001**
Face Mask Agent	-1.41	1.04	-3.47 – 0.66	-1.35	81.00	0.180
Face Mask Participant	-8.51	5.76	-19.96 – 2.94	-1.48	86.23	0.143
SPIN	0.52	0.32	-0.12 – 1.16	1.62	79.93	0.109
Face Mask Agent x Face Mask Participant	1.27	0.46	0.36 – 2.18	2.78	81.00	**0.007**
Face Mask Agent x SPIN	-0.03	0.05	-0.14 – 0.08	-0.57	81.00	0.573
Face Mask Participant x SPIN	0.09	0.29	-0.48 – 0.66	0.33	80.00	0.744

Hypochondria (Whiteley Index)

Table S8. Linear mixed effect model including the individual measure of hypochondria (Whiteley Index) for the analysis of minimal distance in the detour path trials. Model equation: minimal distance ~ 1 + Face Mask Agent Face Mask Participant + Face Mask Agent* Whiteley Index + Face Mask Participant* Whiteley Index + (1+ Face Mask Agent| Subject)*

Predictors	*Estimates*	*SE*	*95% CI*	*t*	*df*	*p*
Intercept	129.69	4.20	121.33 – 138.04	30.88	80.48	**<0.001**
Face Mask Agent	-1.62	0.68	-2.98 – -0.25	-2.36	81.00	**0.021**
Face Mask Participant	-6.57	3.96	-14.44 – 1.29	-1.66	89.03	0.100
Whiteley Index	1.17	1.59	-1.99 – 4.33	0.74	80.36	0.464
Face Mask Agent x Face Mask Participant	1.22	0.46	0.32 – 2.13	2.68	81.00	**0.009**
Face Mask Agent x Whiteley Index	-0.16	0.26	-0.68 – 0.35	-0.62	81.00	0.535
Face Mask Participant x Whiteley Index	0.18	1.42	-2.65 – 3.00	0.12	80.00	0.902

Sensitivity to Reward

Table S9. Linear mixed effect model including the individual measure of Sensitivity to Reward (SR) for the analysis of minimal distance in the detour path trials. Model equation: minimal distance ~ 1 + Face Mask Agent Face Mask Participant + Face Mask Agent* SR + Face Mask Participant* SR + (1+ Face Mask Agent| Subject)*

Predictors	*Estimates*	*SE*	*95% CI*	*t*	*df*	*p*
Intercept	137.72	7.59	122.61 – 152.83	18.14	79.61	**<0.001**
Face Mask Agent	-3.61	1.20	-5.99 – -1.23	-3.01	80.00	**0.003**
Face Mask Participant	-9.03	6.90	-22.75 – 4.70	-1.31	83.77	0.195
Sensitivity Reward	-0.61	0.73	-2.06 – 0.85	-0.83	79.84	0.409
Face Mask Agent x Face Mask Participant	1.16	0.45	0.27 – 2.05	2.60	80.00	**0.011**
Face Mask Agent x Sensitivity Reward	0.16	0.11	-0.06 – 0.39	1.43	80.00	0.158
Face Mask Participant x Sensitivity Reward	0.26	0.65	-1.05 – 1.56	0.39	79.00	0.696

Sensitivity to Punishment

Table S10. Linear mixed effect model including the individual measure of Sensitivity to Punishment (SP) for the analysis of minimal distance in the detour path trials. Model equation: minimal distance ~ 1 + Face Mask Agent Face Mask Participant + Face Mask Agent* SP + Face Mask Participant* SP + (1+ Face Mask Agent| Subject)*

Predictors	*Estimates*	*SE*	*95% CI*	*t*	*df*	*p*
Intercept	126.52	7.35	111.89 – 141.15	17.21	80.00	**<0.001**
Face Mask Agent	-1.38	1.17	-3.70 – 0.94	-1.18	80.00	0.241
Face Mask Participant	-8.55	6.64	-21.76 – 4.66	-1.29	84.49	0.201
Sensitivity Punishment	0.51	0.65	-0.79 – 1.80	0.78	79.94	0.439
Face Mask Agent x Face Mask Participant	1.17	0.45	0.27 – 2.07	2.58	80.00	**0.012**
Face Mask Agent x Sensitivity Punishment	-0.06	0.10	-0.27 – 0.14	-0.60	80.00	0.551
Face Mask Participant x Sensitivity Punishment	0.19	0.58	-0.96 – 1.33	0.32	79.00	0.748
